# Serum protein concentration and amino acid profile of HIV/HBV co-infected subjects on HAART in Plateau State, Nigeria

**DOI:** 10.4314/ahs.v22i1.51

**Published:** 2022-03

**Authors:** Chidi Uzoma Igwe, Emmanuel Emagba Ewuga, Cosmas Onyedikachi Ujowundu, Ignatius Oparaji Onyeocha, Viola Adaku Onwuliri

**Affiliations:** 1 Department of Biochemistry, Federal University of Technology Owerri, Nigeria; 2 Department of Biotechnology, Federal University of Technology Owerri, Nigeria

**Keywords:** Total protein, albumin, globulin, amino acid, viral infection

## Abstract

**Background:**

Human immunodeficiency virus (HIV) and hepatitis B virus (HBV) are currently two important blood-borne human pathogens of major public health concern with high prevalence rates in Africa.

**Objectives:**

The study assessed the impact of HIV and HBV mono- and co-infections on serum total protein, albumin, globulin fractions and plasma free amino acids concentrations.

**Methods:**

This was a cross-sectional study on adult (25 – 64 years old) patients on Highly Active Antiretroviral Therapy attending AIDS Preventive Initiative in Nigeria Centre, Jos University Teaching Hospital, Plateau State, Nigeria. It involved 80 subjects; 20 HIV/HBV co-infected, 20 each of HIV and HBV mono-infected controls, and 20 seronegative controls.

**Results:**

Significant (p<0.05) increases in total protein and gamma globulin but a reduction in albumin concentrations were observed in the HIV/HBV co-infected group. Similarly, significant (p<0.05) increases in alpha-1 and alpha-2 globulin concentrations were observed in the mono- and co-infected groups compared to the seronegative control group. There were significant (p<0.05) increases in the glucogenic, aromatic and branched-chain amino acid concentrations of the HIV/HBV co-infected subjects.

**Conclusion:**

The study suggests prognostic importance of alpha and gamma globulin fractions of serum protein as well as amino acid profile in the management of HIV/HBV co-infection.

## Introduction

Human immunodeficiency virus (HIV) and hepatitis B virus (HBV) are currently two important blood-borne human pathogens of major public health concern. Given the improved life expectancy of people living with HIV due to antiretroviral therapy (ART), liver disease-associated mortality has emerged as the leading cause of death among such population. Of all the possible causes of liver-related deaths, HBV co-infection has become one of the important burden among HIV-positive individuals in post-ART era^1^.

HIV and HBV are transmitted through common routes; therefore, simultaneous infection with both viruses is common. Several reasons highlight the role of HIV infection in the natural history of hepatitis. These include increased number of persistent infection, higher HBV DNA level, lower rate of HBV antigen clearance, progression to liver cirrhosis, liver-related mortality, and increased risk of hepatocellular carcinoma at lower cluster of differentiation 4 (CD4) T cell count^2^. HIV/HBV co-infection is a growing concern because apart from increasing toxicity to antiretroviral medications, co-infected patients have higher levels of HBV replication, lower rates of spontaneous resolution of the HBV infection, and higher risk of reactivation of previous infections^3^. Co-infection with HIV modifies the natural history of HBV infection by increasing the rate of HBV chronic infection, lowering the rate of hepatitis B virus surface antigen (HBsAg), hepatitis B envelope antigen (HBeAg) seroconversion and increasing HBV replication. The deleterious effect of HIV leads to more rapid progression towards end-stage liver diseases (liver cirrhosis and hepatocellular carcinoma) and higher risk for liver-disease related mortality in HIV/HBV co-infected individuals as compared to those infected with HBV only. Thus, in the presence of HIV, management of HBV becomes complicated greatly^1^ and vice versa.

HIV and other infections may have an influence on serum protein patterns of patients. There may also be ethnic and racial differences in the effects of HIV on immunoglobulin production^4^. Separation of serum protein fractions is very important for the diagnosis of different diseases. The pattern of serum protein in normal or disease conditions depends on the fractions of two major types of protein: albumin and globulins. Albumin, the major protein component of serum, is produced by the liver under normal physiologic conditions, which HBV and even HIV infection may affect greatly. Globulins, on the other hand, comprise a much smaller fraction of the total serum protein content, but whose concentration does greatly vary in the presence of infection. The subsets of these proteins and their relative quantity are the primary focus of the interpretation of serum protein electrophoresis (SPE). It has been reported that hepatitis viruses and HIV infections cause irregular alterations in the electrophoretic protein patterns in serum of patients^5^. These alterations are represented at the protein level by disappearance of normal protein bands and / or appearance of abnormal unique bands. This is likely to be caused, among other factors, by hyperactivation of B cells due to chronic antigenic stimulation by antigens of HIV, other viruses or other opportunistic infections^4^.

Metabolic profiling data can be used to define biological status for diagnostic purposes through multivariate analysis as metabolic changes alter the amino acid balance in patients with various diseases. Reports have demonstrated that multivariate analysis of plasma free amino acid profiles is a promising and versatile method for diagnosing various diseases^6^. At the same time, metabolic changes in patients with various pathological conditions lead to alterations in their amino acid profiles. The concentration of amino acids in the extracellular medium was found to be critical in the life cycle of some viruses^6^. Observational studies indicate that amino acid imbalances are related to increased risk of rapidly progressing virus infections through changes of the protein homeostasis. Amino acid profiles may be useful as early biomarkers of physiological disturbances and disease progression^7^. Thus, we assessed the amino acid compositions of HIV and HBV mono- and co-infected subjects as possible indices of biochemical changes during these infections. Furthermore, viral particles during infection are packaged from free amino acids of the host cell. Thus, knowledge of changes in free amino acids composition and serum protein fractions distribution in the host cell and tissue, as well as their modifications by infection, should be of immense importance to healthcare providers. Therefore, the present study was designed to assess the impact of HIV and HBV mono- and co-infections on serum total protein, serum protein fractions and plasma free amino acid compositions of seropositive subjects on Highly Active Antiretroviral Therapy (HAART).

## Materials and methods

### Study area

This is a cross-sectional study among patients on Highly Active Antiretroviral Therapy (HAART) attending AIDS Preventive Initiative in Nigeria (APIN) Centre, Jos University Teaching Hospital (JUTH), Plateau State, Nigeria from March to October, 2016.

### Study design and population

The study is a case control study targeted at adult population attending APIN Centre, Jos. It involved 80 subjects made up of 20 HIV/HBV co-infected, 20 each of HIV and HBV mono-infected controls, and 20 HIV and HBV seronegative normal controls. They were randomly recruited and enrolled into the study. All the subjects were screened for HIV, HBV and HCV using rapid test kits [ALERE Determine HIV-1/2 Ag/Ab Combo rapid test strip (Alere Medical Co., Ltd, Japan), Grand medical HBsAg Rapid test strip (Grand Medical Diagnostics, USA) and Swe-Care HCV rapid test strip (Swe-Care Diagnostic, USA) respectively].

The study population comprised of subjects aged between 16 and 64. The co-infected and HIV mono-infected subjects were drawn from patients that had been recruited in the JUTH/APIN programme and were being monitored on HAART. However, the HBV mono-infected subjects were HBV naive individuals referred for HBV viral load test and are yet to commence any form of medication, while the normal control subjects were apparently healthy HIV and HBV seronegative individuals randomly selected from the hospital environment in the city of Jos, Nigeria.

### Inclusion criteria

The characteristics that made the individuals eligible for the studies included patients with HIV and HBV co-infection that were on HAART; patients with HIV mono infection, that were on HAART; patients with HBV mono-infection but not on medication; apparently healthy individuals that are seronegative to both HIV and HBV, and subjects that willingly consented to be part of the study.

### Exclusion criteria

Subjects with incomplete records and those positive for Hepatitis C virus (HCV) or Human papiloma virus (HPV) were excluded from the study. Also excluded from the study were patients diagnosed of any form of cancer or diabetes. Similarly, alcoholics and subjects that did not willingly give their consent were disqualified from the study.

### Ethical approval

Ethical approvals for the study were obtained from the Ethics Committee of JUTH, and the Site and Research Coordinator of APIN Centre. Participation was voluntary and written informed consent was obtained from each participant. Demographic data of subjects was obtained from APIN clinical records and via administration of questionnaire.

### Sample collection

Blood sample (10 ml in EDTA vacutainer for plasma and plain vacutainer for serum) was drawn from each subject at phlebotomy unit of the hospital. For serum, the blood sample was allowed to coagulate and then centrifuged (Eppendorf 5702, Germany) at a speed of 2500 rpm for 15 min. Sera were separated in a Biosafety cabinet (Nu'aire, NU-425-400E) in a cell separation unit and aliquots for different assays were kept frozen at - 80° C for 24 hours in preparation for analysis.

### Biochemical analysis

Serum protein electrophoresis (SPE) was carried out using Helena Electrophoresis System (Helena Laboratories, Beaumont, TX; Sebia, Norcross, GA.USA) according to manufacturer's instructions. It was performed on Tittan cellulose acetate plates from Helena laboratories at pH 8.6. Quantitation of the different fractions of proteins was estimated by scanning at 525 nm using a densitometer.

The plasma amino acid profile was determined using an auto amino acid analyzer (L-8800; Hitachi, Tokyo, Japan). Briefly, the plasma (0.5 ml) was mixed with 4% sulfosalicylic acid (1.5 ml) and centrifuged at 26,900 x g for 15 min. Then, 1 ml of the supernatant in a vial was applied to the analyzer, which automatically sucks 0.02 ml for amino acid analysis with spectrophotometric detection after post-column reaction with ninhydrin reagent.

The ammonia concentration was measured using an automatic biochemical analyzer (CX9, Beckman, USA) via enzymatic ammonia determination method. Briefly, 0.5 ml of plasma was mixed with 10 µl of L-glutamate dehydrogenase, α-oxoglutarate and NADPH solution. The mixture was incubated for 5 min at 25°C and 1 ml of the supernatant applied to the biochemical analyzer, which automatically measures change in reactant/product concentration spectrophotometrically.

### Statistical analysis

Data analysis was carried out using SPSS 20.0 for Windows software (SPSS Inc., Chicago, IL, USA). Results were expressed as mean ± standard deviation. Comparison between mean values was made by one-way analysis of variance (ANOVA) and least significant difference (LSD) test. The limit of statistical significance was set at p≤0.05.

## Results

**Figure 1:**
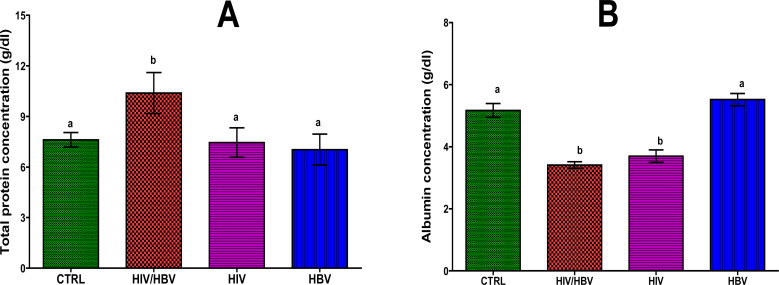
Total protein (A) and albumin (B) concentrations of HIV and HBV mono- and co-infected subjects. Bars are mean ± standard deviation. Bars with different alphabet letter per graph are statistically significant (p<0.05). CTRL, Normal control; HIV/HBV, co-infected group; HIV Mono, HIV mono-infected; HBV, HBV mono-infected.

Figure 1A shows serum total protein concentrations of the subjects. The result indicates significantly higher (p< 0.05) serum total protein concentration in the co-infected patients (12.39 ± 1.22 g/dl) than those of the mono-infected subjects (HIV, 7.45 ± 0.88 g/dl and HBV, 5.03 ± 0.92 g/dl) and the seronegative control (7.61 ± 0.43 g/dl). However, there was no significant difference (p> 0.05) between the total protein concentration of the HIV mono-infected and the seronegative control subjects. Figure 1B shows significant decrease (p < 0.05) in the albumin concentration of the co-infected subjects (3.41 ± 0.49 g/dl) compared to seronegative control (5.17 ± 1.00 g/dl) and HBV mono-infected (5.52 ± 0.89 g/dl) subjects. However, there was no significant difference (p < 0.05) in the albumin concentration between the HIV/HBV co-infected and the HIV mono-infected (3.70 ± 0.90 g/dl) subjects, and between the seronegative control and HBV mono-infected subjects.

**Figure 2:**
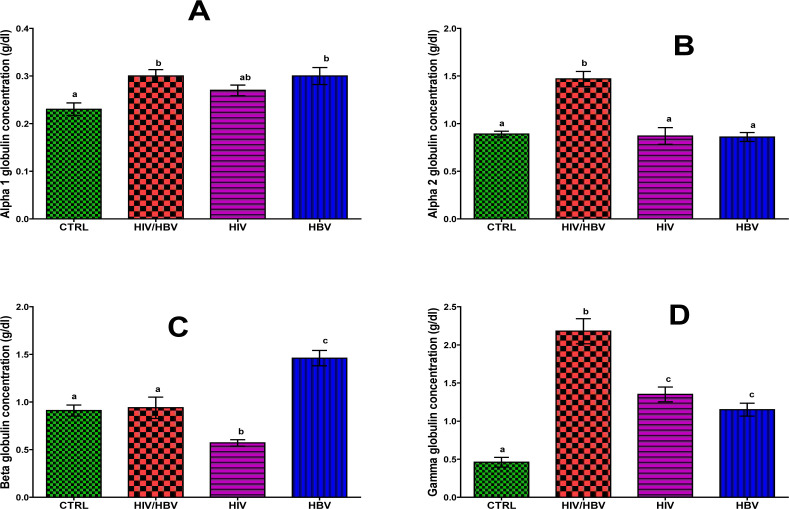
Globulin fractions concentrations of HIV and HBV mono- and co-infected subjects. Bars are mean ± standard deviation. Bars with different alphabet letters per graph are statistically significant (p<0.05). CTRL, Normal control; HIV/HBV, co-infected group; HIV Mono, HIV mono-infected; HBV, HBV mono-infected.

The co-infected subjects showed a higher significant increase (p< 0.05) in alpha 1 globulin fraction (0.30 ± 0.06 g/dl) than seronegative control subjects (0.23 ± 0.06 g/dl), but no significant difference (p> 0.05) with the mono-infected subjects (HIV, 0.27 ± 0.05 g/dl and HBV, 0.30 ± 0.08 g/dl) (Figure 2A). Similarly, alpha 1 concentration of the seronegative control subjects showed no significant difference (p> 0.05) with that of the HIV mono-infected subjects.

Figure 2B presents significantly higher (p< 0.05) alpha 2 globulin concentration in the co-infected subjects (1.47 ± 0.35 g/dl) than those of the mono-infected (HIV, 0.87 ± 0.39 g/dl and HBV, 0.86 ± 0.21 g/dl) and the seronegative control (0.89 ± 0.14 g/dl) subjects. However, there was no significant difference (p> 0.05) in alpha 2 concentration of the mono-infected subjects and the seronegative control.

Similarly, there was significantly higher (p< 0.05) beta globulin concentration in the co-infected subjects (0.94 ± 0.50 g/dl) than in the HIV mono-infected subjects (0.57 ± 0.15 g/dl), but was significantly lower (p< 0.05) when compared to the HBV mono-infected subjects (1.46 ± 0.36 g/dl) (Figure 2C). However, there was no significant difference (p> 0.05) in the beta globulin concentration of the HIV/HBV co-infected in comparison with the seronegative control (0.91 ± 0.26 g/dl) subjects.

Figure 2D shows significantly higher (p< 0.05) gamma globulin concentration in the co-infected subjects (2.18 ± 0.74 g/dl) than those of the mono-infected (HIV, 1.35 ± 0.43 g/dl and HBV, 1.15 ± 0.38 g/dl) and the seronegative control (0.46 ± 0.29 g/dl) subjects. However, there was no significant difference (p> 0.05) in the gamma globulin concentration of the mono-infected subjects.

Table 1 shows the essential amino acid profile of the study groups. There were significantly higher (p< 0.05) concentrations of plasma total essential amino acids in the HIV/HBV co-infected (1143.45 ± 36.71 mg/dl) and HIV mono-infected (1130.19 ± 34.20 mg/dl) subjects compared to the HBV mono-infected (506.50 ± 19.44 mg/dl) and the normal control (429.80 ± 14.99 mg/dl) subjects. Generally, the infected subjects had significantly higher (p< 0.05) essential amino acid compositions than the uninfected, with the co-infected apparently having the highest distribution of these amino acids.

**Table 1:** Essential amino acid concentrations (mg/dl) of HIV and HBV mono- and co-infected subjects

Essential amino acids (mg/dl)	Normal control (n=20)	HIV/HBV co-infected (n=20)	HIV mono-infected (n=20)	HBV mono-infected (n=20)
Thr	34.67 ± 14.37^c^	178.05 ± 63.60^a^	106.57 ± 22.29^a^	99.36 ± 22.83^b^
Val	109.61 ± 23.06^c^	155.74 ± 31.85^a^	127.10 ± 32.56^ac^	24.56 ± 7.23^b^
Met	88.71 ± 34.41^b^	120.69 ± 30.08^a^	87.23 ± 22.14^b^	97.95 ± 27.29^ab^
Leu	39.08 ± 7.16^b^	114.32 ± 34.28^a^	125.24 ± 39.46^a^	64.62 ± 29.65^b^
Ile	39.43 ± 13.08^c^	160.17 ± 29.56^a^	160.36 ± 28.19^a^	114.98 ± 40.79^b^
Phe	33.91 ± 7.62^b^	156.28 ± 47.36^a^	191.29 ± 45.45^a^	41.59 ± 8.70^b^
Lys	36.55 ± 8.18^b^	156.64 ± 35.73^a^	132.49 ± 29.70^a^	29.95 ± 7.96^b^
His	47.84 ± 12.06^c^	101.56 ± 21.23^a^	199.91 ± 53.78^b^	33.49 ± 11.08^c^

**Total**	**429.80 ± 14.99^c^**	**1143.45 ± 36.71^a^**	**1130.19 ± 34.20^a^**	**506.50 ± 19.44^b^**

Values are mean ± standard deviation. Values with different superscript letter(s) per row are statistically significant (p<0.05).

Similarly, there were significantly higher (p< 0.05) plasma total non-essential amino acid profile in the HIV/HBV co-infected and HIV mono-infected (1210.64 ± 39.85 mg/dl and 1233.51 ± 33.83 mg/dl respectively) subjects compared to the HBV mono-infected (421.60 ± 11.82 mg/dl) and the normal control (390.95 ± 13.05 mg/dl) subjects (Table 2). A trend similar to the essential amino acid composition among the subjects was observed, in which the infected subjects had significantly higher (p<0.05) non-essential amino acid compositions than the uninfected, but with the HIV mono-infected apparently having the highest distribution of the non-essential amino acids. Table 2 also shows a significantly higher (p< 0.05) plasma ammonia concentration in the HIV/HBV co-infected (166.87 ± 23.81 mg/dl) and HIV mono-infected (181.77 ± 28.05 mg/dl) subjects compared to the HBV mono-infected (39.53 ± 9.63 mg/dl) and the normal control (37.04 ± 11.39 mg/dl) subjects.

**Table 2:** Non-essential amino acids and ammonia concentrations of HIV and HBV mono- and co-infected subjects

Non-essential amino acids (mg/dl)	Normal control (n=20)	HIV/HBV co- infected (n=20)	HIV mono- infected (n=20)	HBV mono- infected (n=20)
Asp	36.54 ± 11.25^b^	99.44 ± 33.76^a^	89.87 ± 28.63^a^	111.80 ± 28.61^a^
Ser	34.69 ± 12.53^c^	142.83 ± 35.31^a^	63.06 ± 28.30^b^	63.38 ± 11.55^bc^
Glu	32.00 ± 8.19^b^	134.75 ± 34.07^a^	205.60 ± 54.84^a^	31.04 ± 8.39^b^
Ala	30.54 ± 8.65^b^	165.28 ± 41.58^a^	94.41 ± 23.86^a^	30.14 ± 8.36^b^
Gly	30.37 ± 11.86^b^	201.73 ± 89.42^a^	158.05 ± 36.30^a^	30.55 ± 9.17^b^
Cys	112.43 ± 27.61^a^	137.03 ± 36.26^a^	166.45 ± 39.92^a^	36.71 ± 9.33^b^
Tyr	31.28 ± 7.79^b^	99.09 ± 27.88^a^	199.12 ± 39.74^a^	40.37 ± 7.98^b^
Arg	52.21 ± 18.92^b^	98.43 ± 23.74^a^	165.70 ± 33.34^a^	37.55 ± 11.89^b^
Pro	30.89 ± 10.69^b^	132.06 ± 36.61^a^	91.25 ± 19.50^a^	40.06 ± 11.12^b^

**Total**	**390.95 ± 13.05^c^**	**1210.64 ± 39.85^a^**	**1233.51 ± 33.83^a^**	**421.60 ± 11.82^b^**

NH_3_	37.04 ± 11.39^b^	166.87 ± 23.81^a^	181.77 ± 28.05^a^	39.53 ± 9.63^b^

Values are mean ± standard deviation. Values with different superscript letter(s) per row are statistically significant (p<0.05)

## Discussion

Protein and albumin determinations are frequently included in the routine laboratory evaluation of HIV and HBV infected patients, mainly because of the negative effects of both infections on the liver. Albumin makes up more than half of the total protein within the blood, while globulins make up the remainder^8^. Results of the present study showed that HIV/HBV co-infection was significantly associated with increase in total protein and decrease in albumin. The observed increase in total protein may be caused by dehydration, metabolic alteration and most possibly from increased globulin fraction^9^–^10^. Meanwhile, we observed a non-significant decrease in total protein concentration of the HBV and HIV mono-infected patients. This decrease could be attributed to either increased losses and/or catabolism or as a result of reduction in intake and/or absorption due to sores in the mouth, pharynx and/or oesophagus, fatigue, depression and side effects of medications as is common in HIV infection^11^. Most blood proteins, especially albumin, are solely synthesized by the liver. On the other hand, anti-viral drugs like other xenobiotics are metabolized by the liver. A compromise of the liver function will directly affect the outcome of blood total protein and albumin concentrations. Thus, our obsevations in these parameters is in tandem with earlier reports that natural history of HIV/HBV co-infection show liver-related mortality that is 19 and 8 times higher than in individuals with HBV and HIV mono-infections respectively^12^.

On the other hand, we observed a significant decrease (p< 0.05) in albumin concentration of the co-infected subjects which suggests that either HIV infection or the medication (HAART) is associated with hypoalbuminemia considering the corresponding observation of no significant difference in the albumin concentration of the HIV/HBV co-infected subjects and the HIV mono-infected subjects. Serum albumin level is considered one of the prognostic markers of HIV infection, as it has been shown that a low level of serum albumin, after seroconversion is associated with faster HIV disease progression^13^. Serum albumin concentration therefore, appears to be a sensitive predictor of preclinical disease and disease severity^9^. Similarly, low albumin typically occurs at advanced stages of HIV disease and reflects poor nutritional status, catabolic state of chronic disease evidenced by significant weight loss^14^. Hypoalbuminemia in HIV infection has been attributed to inadequate nutrient intake and metabolic alteration.

Elevation in total protein and globulin fractions can also occur in otherwise healthy HIV-positive individuals. It probably reflects a generalized polyclonal gammopathy, with increased antibody production, which is an attempt on the part of the immune system to compensate for cellular immunodeficiency. This might also explain our observation of the HIV mono-infected groups having similar total protein concentration with the seronegative control group. Although, serum total protein estimation has limited diagnostic importance when compared to albumin because of the compensatory increases in the globulins during infections, its relevance in the evaluation of patients with some clinical conditions such as malnutrition, malignancy, renal and liver diseases and immune disorders cannot be ignored^14^.

Globulins are a group of proteins within the blood that are produced by the liver and the immune system^8^. Serum globulins comprised of alpha-1 (10%; mainly alpha-1 antitrypsin), alpha-2 (30%; alpha 2 macroglobulin and haptoglobin), beta globulins (34%; transferrin and complement components C3, C4, C5) and gamma globulins (46%; mostly immunoglobulins)^15^. Globulins serve multiple different functions in the body, which include their roles as immunoglobulins, enzymes, carrier proteins and complement^8^.

Among the co-infected subjects, there was an elevation in alpha-1 and alpha-2 globulin concentrations when compared to the seronegative control. O'Connell et al reported that malignancy and acute inflammation (resulting from acute-phase reactants) can increase the alpha-1 protein band, while the alpha-2 component is increased as an acute-phase reactant^16^. HIV infection is characterized by inflammation, defect in cellular immunity and cellular immune activation with a depression ratio of helper Tlymphocyte to suppressor T-lymphocyte^17^. Alpha-1 microglobulin on the other hand is actively produced and secreted by T and B lymphocytes and by the liver^18^. The rise in serum levels of alpha-1 in both HIV/HBV co-infected and HBV mono-infected group, compared to the HIV mono-infected and seronegative subjects suggests that alpha-1 can be used to monitor the level of inflammation and cellular immune activation associated with disease progression in HIV/HBV seropositive subjects, but not in HIV mono-infected subjects on HAART. Meanwhile, we observed no significant difference in the alpha-1 and alpha-2 concentrations of the seronegative control and HIV mono-infected subjects. This observation could be attributed to the effect of HAART. This is in agreement with reported positive association between CD4+ T-cell count and alpha-1 microglobulin in non-ART HIV patients but not, in ART group^9^. It was suggested that this could be due to reduction in viral replication by the drugs resulting in the improvement of health. A similar finding of reduced level of globulin fraction after initiation of antiretroviral therapy had also been earlier documented^19^. Elevation in globulin fraction is thus a characteristic of chronic inflammatory condition generally created by viral infection, and this could explain the observed increase in alpha-1 and beta globulin levels of HBV mono-infected subjects.

The beta fraction of globulins consists mostly of transferrin (beta-1) which is elevated in severe iron deficiency^8^ and beta-2 which contains beta-lipoprotein. Immunoglobulin A (IgA), immunoglobulin M (IgM), and sometimes immunoglobulin G (IgG), along with complement proteins also can be identified in the beta fraction^16^. Although, our study did not distinguish between beta-1 and beta-2 microglobulin fractions, but we observed that serum beta microglobulin showed a significantly higher concentration in the HBV mono-infected and HIV/HBV co-infected subjects than that of the HIV mono-infected subjects. This could be a direct reflection of HBV burden on HIV. Beta-2 microglobulin (β2M) is an amino acid peptide^20^, a component of the class I major histocompatibility leukocyte antigen molecule (HLA) which is present on the surface of almost all nucleated host cells. Significant high serum levels of β2M are said to be encountered in a variety of inflammatory and neoplastic diseases, in acute and chronic hepatitis and in the presence of hepatic cirrhosis^21^. It appears that an increase in the β2M concentration in serum is an index of an on-going inflammatory changes in the liver. A high serum level of β2M have been detected in many infectious diseases including HCV^22^. An increase in β2M serum levels has also been associated with AIDS development and death, and higher concentrations are found in patients with early progression to AIDS^23^. The β2M is produced predominately by B lymphocyte, but upon immune system activation both T and B lymphocytes actively release beta-2 microglobulin into the circulation^24^. Therefore, β2M plays a key role in influencing the immune response to viral infections^25^. Hence, β2M in serum can aid in the clinical assessment of activation of cellular immune system. Ezugwu et al. reported an increased β2M concentration in HIV seropositive group, stating that the β2M concentration of non-ART group showed a higher significant increase (p<0.05) when compared with ART group^9^. This suggests that β2M continues to rise as the disease progresses to AIDS. Thus, the decreased β2M observed among the HIV mono-infected group and the normal level of β2M in the HIV/HBV co-infected subjects suggests the positive impact of HAART in preventing the progression of the HIV infection to AIDS.

Chronic infections such as HIV infection, liver diseases, and lymphoma have been reported to be the most common causes of hypergammaglobulinemia^9^. Increased globulin concentration usually occurs in the gamma-globulin fraction (or immunglobulins) with a corresponding increase in the total protein concentration. While the T-cell compartment reconstitution is monitored routinely in clinical practice in patients started on HAART, B-cell immune reconstitution is not^15^. Thus, our assessment of the B-cell reconstitution may have clinical implications. Our result showed that the HIV/HBV co-infected subjects had elevated gamma globulin concentration in comparison with the uninfected control, HIV and HBV mono-infected groups. Chronic HIV infection induces hypergammaglobulinemia via polyclonal B-cell activation and spontaneous secretion of immunoglobulins by abnormally activated B-cells^26^. Similarly, serum gamma globulin elevation in association with chronic hepatitis B is a well-known phenomenon^18^. Under such circumstances, immunoglobulin G (IgG) against viral or bacterial antigen is abundantly synthesized, which leads to hypergammaglobulinemia^27^. Thus, elevation of serum IgG level is a clinical feature of chronic liver disease and is highly associated with disease progression^18^. Despite report on HAART-induced improvement in immune function, including B-cell count and function, the observed high gamma globulin clearly reflects the impact of HBV on HIV and vice-versa. Also, the elevated gamma globulin concentrations observed among the HIV- and HBV- mono-infected subjects indicate that it is a clinical feature of IgG against viral infection^26^, and thus an indication of potential prognostic relevance of gamma globulin assessment in HIV/HBV infection.

Plasma amino acids and their derivatives have a close relationship with energy, protein metabolism and body nutrition. Any fluctuation in their normal levels can indicate diseased status or some special physiological condition. They reflect not only the nutritional status and metabolic abnormalities, but can also be used as prognostic markers of various diseases^28^. Free amino acids serve as substrates for protein synthesis, gluconeogenesis, ureagenesis and other catabolic processes^29^. Several diseases, especially consuming illnesses such as liver and metabolic diseases, can induce specific patterns in plasma amino acids, with increased concentrations of phenylalanine, glutamate, and arginine. Likewise, protein metabolism is commonly altered with severe illness, leading to changes in blood amino acid concentrations^30^. These changes have been shown to have prognostic value in HIV infection^31^. Amino acids are also important for T-cell functions, serving as both a source of energy and as biosynthetic precursors. It has been documented that glutamine uptake by T-lymphocytes increases upon their activation, and glutamine is well-known to be a major fuel substrate for immune cells^32^.

Similar to other illnesses, HIV/HBV co-infection causes metabolic and net catabolic changes through processes including increased metabolic rate, malabsorption, anorexia and wasting syndrome^33^. A small number of studies in the pre-combination antiretroviral therapy (cART) era showed that adults with HIV infection and AIDS have decreased plasma concentrations of amino acids^31^. Similarly, amino acid metabolism in patients with hepatitis B virus infection is significantly altered. These changes manifest as an increase in concentration of aromatic amino acids (AAAs) and a decrease in concentration of branched-chain amino acids (BCAAs). BCAAs have been applied to the diagnosis of viral hepatitis B and as an adjuvant therapy for liver disease^33^. Little information is available on the plasma amino acid patterns in patients with HIV/HBV co-infection disease, or in patients receiving HAART.

Our study suggests that significant changes occur in plasma concentrations of amino acids in patients with HIV/HBV co-infection receiving HAART. The study demonstrated that the plasma concentrations of most essential amino acids (threonine, leucine, isoleucine, phenylalanine, lysine and histidine) and many other non-essential amino acids (arginine. proline, tyrosine, glycine, alanine, aspartate, serine and glutamine) in HIV/HBV co-infected and HIV mono-infected patients receiving HAART were significantly higher than those of the seronegative control.

The amino acids threonine, glycine, alanine, and aspartate are major precursors for gluconeogenesis and their elevation therefore suggests that gluconeogenesis is generally impaired in HIV/HBV co-infection. It was suggested that HIV-infected patients have a selective deficiency in threonine^34^. This selective threonine deficiency could arise from an activation of the catabolism of threonine and/or synthesis of threonine-rich proteins. However, our study recorded an elevation in plasma threonine in the infected groups, compared to the control group suggesting low rate of gluconeogenesis in the infected subjects. This observation is in line with the fact that viral infections trigger metabolic alterations in host cells that support the energetic and biosynthetic demands of viral replication^28^. Glycine, a precursor of glutathione, exerts indirect protective effect against free radical-induced liver injury via synthesis of glutathione^35^. Elevation in glycine and alanine among HAART receiving groups (HIV/HBV co-infected and HIV mono-infected) further demonstrates lowered activity of gluconeogenesis and the struggle to generate more glutathione in this group of subjects. The present result also showed that aspartate is elevated among the infected groups compared to the seronegative control group. High plasma aspartate concentrations were found in patients with chronic active hepatitis, primary biliary cirrhosis, and cryptogenic cirrhosis^36^. Aspartate serves as a nitrogen donor in the urea cycle; if intracellular values were low, impaired ammonia detoxification could result. Aspartate may also serve as an excitatory neurotransmitter in the brain, so that low intracelllar levels could result in impaired cerebral function^37^. Similarly, serine another gluconeogenic amino acid, was also increased in the HIV/HBV co-infected group compared to the seronegative control, further alluding to the suggested decrease in gluconeogenesis in the co-infected group.

The amino acids valine, leucine, and isoleucine are all essential amino acids, known as the branched chain amino acids (BCAAs). They are primarily catabolised via the skeletal muscle and kidneys^36^, where they react with alpha-ketoglutaric acid and are converted to glutamine by transamination. Muscle has a substantial ability to catabolise BCAAs producing glutamine and alanine, and oxidizing the branched-chain keto-acids^38^. Our result showed that concentrations of BCAAs are increased in the HIV/HBV co-infected group. The observed increases in the BCAAs indicate that ketogenesis is impaired in the infected groups (HIV/HBV co-infected, HIV mono-infected and HBV mono-infected) leading to significant accumulation of valine, iso-leucine and leucine. Although, low concentrations of circulating BCAAs has been noted as a hallmark of liver disease^39^, the increased BCAAs concentration observed in the present study reflects either a low level of hepatocellular damage or the beneficial effects of HAART.

Meanwhile, in the HBV mono-infected group, the plasma concentration of valine was significantly reduced indicating liver disease, based on the observation that, during hepatic failure, plasma concentrations of BCAAs decrease^33^. In humans, the liver is of major importance in amino acid transamination. Its ability to use the three branched chain amino acids, however, is limited since they are catabolised via the skeletal muscle^40^. The capacity of the extra-hepatic tissues to metabolise valine may assume compensatory proportions if liver gluconeogenesis decreases, with the result that plasma concentrations fall. Insulin acts directly on the liver inhibiting gluconeogenesis and peripherally it suppresses muscle output of branched chain amino acids^41^. It was postulated that hyperinsulinaemia which is present in cirrhosis may drive BCAAs to the muscle and the kidneys where they are broken down^36^. Valine is a gluconeogenic amino acid that could enter the Kreb's cycle and proceed towards the production of ATP. When acetyl-CoA is in short supply Kreb's cycle can be fuelled from alternative sources such as valine. This might explain the decreased concentration observed for the amino acid. Therefore, the low levels of valine may be related to a raised circulating insulin concentration. Perhaps none of these explanations serve, however, to explain the reduced circulating valine concentrations in the HBV mono-infected group. Hyperinsulinaemia has been reported in patients with viral hepatitis^36^, but would at best provide only a partial explanation for these findings. On the other hand, our result on leucine and iso-leucine do not reflect this decrease as concentrations of leucine and isoleucine are increased in the HBV mono-infected group though not significantly higher than those of HAART receiving groups. These indicate the need for further studies.

The aromatic amino acids (AAAs) and methionine are catabolised mainly in the liver^36^. Typical changes in plasma amino acid patterns have been found in different studies in patients^42^ and experimental animals in chronic liver failure^43^. The reported changes are mainly increased concentrations of the AAAs and methionine, and decreased concentrations of the BCAAs^39^. Our study recorded a similar pattern of increase in phenylalanine and tyrosine among the HAART receiving groups compare to the HAART naïve groups. These findings related to AAAs confirm reports of previous studies^36^,^42^, which showed an increase in AAAs concentrations. The raised values of the amino acids in HIV/HBV co-infected and HIV mono-infected subjects probably result from impaired hepatic metabolism and portal systemic shunting of blood. Increased gluconeogenesis could also contribute to the abnormal amino acid pattern^37^. Pioneering application of metabolomics technologies has shown that a cluster of amino acids including BCAAs and AAAs was strongly associated with obesity-related insulin resistance^44^. Another explanation suggests that the AAA, phenylalanine and tyrosine, are elevated because the ‘large neutral amino acids’ (valine, isoleucine, leucine, tyrosine, phenylalanine and tryptophan) compete for transport into mammalian cells by the large neutral amino acid transporter, assuming that chronic elevations in BCAAs might impair the transport of AAAs into cells and tissues. In our study, methionine was elevated in the HIV/HBV co-infected and HBV mono-infected groups, also suggesting impaired hepatic metabolism and portal systemic shunting of blood. It has been noted that the hallmark of liver disease is characterized by elevated concentrations of circulating AAAs and methionine^39^.

Plasma concentrations of lysine and its derivative (L-carnitine) have a close relationship with viral and host protein metabolism and they act as indicators to evaluate not only the nutritional status of HIV-infected persons, but also as prognostic markers of the disease^28^. Our result showed that the concentrations of the amino acid, lysine in HIV/HBV co-infected and HIV mono-infected groups were significantly increased compare with the HBV mono-infected and normal control groups. It has been reported that plasma lysine concentration markedly decrease with progression of HIV-infection^28^. At the same time, after initiation of HAART, the blood concentration of lysine significantly increase among HIV-infected individuals^28^. Our results showed that there was evidence for an association between plasma lysine and immunological markers and clinical stages of HIV/HBV co-infection. The study further seems to support the hypothesis that lysine plays important role in the synthesis of the virus proteins and in the initiation of HIV's/HBV's reproduction. It is likely that high concentrations of this essential amino acid in plasma may increase the risk of high HIV RNA levels, subsequent acceleration of immunosuppression and the disease progression.

HIV infection has been reported to cause decreased cysteine concentration^31^. Decrease in cysteine, the rate-limiting substrate for the synthesis of glutathione, may be a factor in triggering the replication of HIV. The present study showed a significantly lower concentration of cysteine in HBV mono-infected group. This could be a direct reflection of pathological processes of infection as well as treatment. No significant increase in cysteine concentration was observed between the HAART receiving and the seronegative control groups, which clearly show the positive impact of HAART in the HIV/HBV co-infected and HIV mono-infected groups.

This study also showed that HIV/HBV co-infected and HIV mono-infected groups have increased concentrations of glutamate, histidine and arginine. These observations are in accordance with studies in experimental animal models of liver failure, and studies in patients suffering from acute liver failure^36^. Glutamate plays a central role in the metabolism of amino acids and ammonia. It is formed in the degradation of arginine, ornithine, proline, and histidine. Increased glutamate concentrations possibly can contribute to the impaired immune reactivity of patients with consuming diseases. Arginine may also play a role as an immune modulator^31^. Arginine is an amino acid that is an intermediary of the urea cycle. Its observed increase in concentration is in agreement with suggested decrease in urea cycle as is the case with increase in histidine concentration which is a glutamate precursor.

Note that we also observed a significantly elevated plasma proline concentration in the HIV/HBV co-infected and HIV mono-infected groups. Proline is used in collagen synthesis, and low concentrations have been suggested to indicate increased collagen production^33^.

In addition to the perturbations observed above, other biochemical abnormalities seem to be present in HIV, HBV and co-infected patients. Hyperammonaemia makes it necessary for the body to find other pathways for nitrogen elimination^37^. Our study showed that HIV/HBV co-infected and HIV mono-infected groups have increased levels of ammonia, suggesting diminished urea production which is in consonance with our observations of increased concentrations of glutamate, histidine and arginne as earlier discussed.

## Conclusion

Increased blood concentrations of total protein, globulin fractions, essential and non-essential amino acid profiles, and observed reduction in serum albumin concentration may be associated with HIV and HBV co-infection. HIV mono-infection compared to HBV gave trends close to the effects of HIV/HBV co-infection. The study suggests prognostic importance of alpha and gamma globulin protein fractions and amino acid profile in the management of HIV/HBV co-infection.
